# Modeling genotype × environment interaction for single and multitrait genomic prediction in potato (*Solanum tuberosum* L.)

**DOI:** 10.1093/g3journal/jkac322

**Published:** 2022-12-08

**Authors:** Jaime Cuevas, Fredrik Reslow, Jose Crossa, Rodomiro Ortiz

**Affiliations:** Departamento de Energía, Universidad Autónoma del Estado de Quintana Roo, Chetumal, Quintana Roo 77019, México; Department of Plant Breeding, Swedish University of Agricultural Sciences (SLU), P.O. Box 190, Lomma SE 23436, Sweden; International Maize and Wheat Improvement Center (CIMMYT), Carretera México-Veracruz Km. 45, El Batán, Texcoco 56237, Edo. de Mexico, Mexico; Colegio de Postgraduados, Montecillos, Edo. de México 56230, México; Department of Plant Breeding, Swedish University of Agricultural Sciences (SLU), P.O. Box 190, Lomma SE 23436, Sweden

**Keywords:** *Solanum tuberosum*, genomic prediction in potato, genomic × environment interaction, multienvironment modeling, multiple trait modeling, single-environment modeling, single-trait modeling

## Abstract

In this study, we extend research on genomic prediction (GP) to polysomic polyploid plant species with the main objective to investigate single-trait (ST) and multitrait (MT) multienvironment (ME) models using field trial data from 3 locations in Sweden [Helgegården (HEL), Mosslunda (MOS), Umeå (UM)] over 2 years (2020, 2021) of 253 potato cultivars and breeding clones for 5 tuber weight traits and 2 tuber flesh quality characteristics. This research investigated the GP of 4 genome-based prediction models with genotype × environment interactions (GEs): (1) ST reaction norm model (*M1*), (2) ST model considering covariances between environments (*M2*), (3) ST *M2* extended to include a random vector that utilizes the environmental covariances (*M3*), and (4) MT model with GE (*M4*). Several prediction problems were analyzed for each of the GP accuracy of the 4 models. Results of the prediction of traits in HEL, the high yield potential testing site in 2021, show that the best-predicted traits were tuber flesh starch (%), weight of tuber above 60 or below 40 mm in size, and the total tuber weight. In terms of GP, accuracy model *M4* gave the best prediction accuracy in 3 traits, namely tuber weight of 40–50 or above 60 mm in size, and total tuber weight, and very similar in the starch trait. For MOS in 2021, the best predictive traits were starch, weight of tubers above 60, 50–60, or below 40 mm in size, and the total tuber weight. MT model *M4* was the best GP model based on its accuracy when some cultivars are observed in some traits. For the GP accuracy of traits in UM in 2021, the best predictive traits were the weight of tubers above 60, 50–60, or below 40 mm in size, and the best model was MT *M4,* followed by models ST *M3* and *M2*.

## Introduction

Genomic prediction (GP) and selection (GS) have changed the paradigm of plant and animal breeding (Meuwissen *et al*. 2001; de los Campos *et al*. 2009; Crossa *et al*. 2010, 2011; Desta and Ortiz 2014). Practical evidence has shown that GS provides important increases in prediction accuracy for genomic-aided breeding (Pérez-Rodríguez *et al*. 2012; Crossa *et al*. 2014, 2017). Additive genetic effects (breeding values) can be predicted directly from parametric and semi-parametric statistical models using marker effects like the ridge regression best linear unbiased prediction (Endelman 2011), or by developing the genomic relationship inear kernel matrix (***G***) to fit the genomic best linear unbiased prediction (GBLUP; VanRaden 2008). Departures from linearity can be assessed by semi-parametric approaches, such as Reproducing Kernel Hilbert Space regression using the Gaussian kernel (GK) or different types of neural networks (Gianola *et al*. 2006; Gianola and Van Kaam 2008; de los Campos *et al*. 2010; González-Camacho *et al*. 2012; Pérez-Rodríguez *et al*. 2012; Gianola *et al*. 2014; Sousa *et al*. 2017).

Standard GP models were extended to multienvironment (ME) data by assessing genomic × environment interaction (GE; Burgueño *et al*. 2012). Jarquín *et al*. (2014) proposed an extension of the GBLUP or random effects model, where the main effects of markers and environmental covariates could be introduced using covariance structures that are functions of marker genotypes and environments. Consistently, GP accuracy substantially increased when incorporating GE and marker × environment interaction (Crossa *et al*. 2017). Cuevas *et al*. (2016) and Sousa *et al*. (2017) applied the marker × environment interaction GS model of Lopez-Cruz *et al*. (2015), but modeled not only through the standard GBLUP but also through a nonlinear GK like that used by de los Campos *et al*. (2010) and a GK with the bandwidth estimated through an empirical Bayesian method (Pérez-Elizalde *et al*. 2015). Cuevas *et al*. (2016) concluded that the higher prediction accuracy of GK models with the GE model is due to more flexible kernels that allow accounting for small, more complex marker main effects and marker-specific interaction effects.

In GP, the training set usually includes a sufficient overlap of lines across environments, so that estimating the phenotypic covariance among environments for modeling GE is sufficient to specify it on the linear mixed model used. When modeling GE, some researchers used the mathematical operation defined by the Kronecker products or direct product (Cuevas *et al*. 2016) that allows operations of 2 matrices of different dimensions. Other authors model GE using the matrix operation named Hadamard products (also known as element-wise products), which is a binary operation between 2 matrices of the same dimensions as the operands (Jarquín *et al*. 2014; Lopez-Cruz *et al*. 2015; Acosta-Pech *et al*. 2017; Perez-Rodriguez *et al*. 2017; Sukumaran *et al*. 2017; Basnet *et al*. 2019). When modeling epistasis, Hadamard products of the additive genomic relationship have mainly been used (e.g. Jiang and Reif 2015; Martini *et al*. 2016; Vitezica *et al*. 2017; Varona *et al*. 2018; Martini *et al*. 2020). However, Burgueño *et al*. (2007) have used Kronecker products for modeling and the estimation of additive, additive × environment interaction, additive × additive epistasis, and additive × additive × environment interactions by means of the coefficient of parentage. In a recent study, Martini *et al*. (2020) gave theoretical proof that both methods lead to the same covariance model when used with some specific design matrices and illustrated how to explicitly model the interaction between markers, temperature, and precipitation.

Traditionally, GP models have evolved from the single-trait (ST) and single-environment prediction (ST-SE) models to ST-ME models including GE. Furthermore, standard GS-assisted plant breeding models are concerned with the assessment of the GP accuracy of a multitrait (MT) measured in a single environment (MT-SE) or MT-MEs. In general, MT GP models have evolved from MT-SE to MT-ME. The MT models are keys for improving prediction accuracy in GS because they offer benefits regarding the ST models when the traits under study are correlated. Most existing models for GP are the ST models although the MT models have several advantages over the ST (Montesinos-López *et al*. 2019). Compared with ST, MT can simultaneously exploit the correlation between cultivar and traits and thus improve the accuracy of GP as they are computationally more efficient than ST (Montesinos-López *et al*. 2019). When the traits are correlated, MT models improve parameter estimates and prediction accuracy as compared to ST models (Schulthess *et al*. 2018; Calus and Veerkamp 2011; Jiang and Jannink 2012; Montesinos-López *et al*. 2016, 2019; He *et al*. 2016). With the continuous growth of computational power, MT models play an increasingly important role in data analysis in plant and animal genomic−aided breeding for selecting the best candidate genotypes.

The use of MT models is not as widespread as the use of ST models because of several factors such as, among others, lack of efficient and friendly software, and not enough computational resources. Likewise, MT models have more complex GEs that make it difficult to assess and achieve MT model assumptions. Furthermore, MT models have more problems of convergence than ST models. Some models have been proposed for MT GP, e.g. MT mixed models and their Bayesian version. Bayesian MT genomic best linear unbiased predictor and MT models under artificial deep neural networks were applied to maize and wheat data sets (Montesinos-López *et al*. 2018, 2019). However, most researchers use MT models to improve prediction accuracy for traits to be predicted (i.e. the prediction set)—which are tedious and time-consuming to measure and have low heritability—by using a few traits (i.e. the training set) with high heritability that are highly correlated with the former prediction set (Jiang and Jannink 2012; Semagn *et al*. 2022).

It is widely recognized that from the statistical and quantitative genetics perspectives, when data on MTs are available, the preferred models are the MT as they can account for correlations between phenotypic traits in the training set because borrowing information from correlated traits increases GP accuracy. Montesinos-López *et al*. (2022) investigated Bayesian MT kernel methods for GP and illustrated the power of linear, Gaussian, polynomial, and sigmoid kernels. The authors compared these kernels with the conventional ridge regression and GBLUP MT models. Montesinos-López *et al*. (2022) showed that, in general, but not always, the GK method outperformed conventional Bayesian ridge and GBLUP MT in terms of GP prediction performance. These authors concluded that the improvement in terms of prediction performance of the Bayesian MT kernel method can be attributed to the proposed model being able to capture nonlinear patterns more efficiently than linear MT models.

Semagn *et al*. (2022) were interested in comparing prediction accuracy estimates of a subset of lines that have been tested for an ST, with a subset of lines that have not been tested for certain proportion traits (MT1, certain cultivars were not tested for any of the traits), and a subset of lines that have been tested for some traits but not for other traits (MT2) across different bread wheat genetic backgrounds for agronomic traits of varying genetic architecture evaluated under conventional and organic management systems, and several host plant resistance traits evaluated in adult plants under standard field management. Their results show that the predictive ability of the MT2 model was significantly greater than that of the ST and MT1 models for most of the traits and populations, except common bunt, with the MT1 model being intermediate between them, thus demonstrating the high potential of the MT models in improving prediction accuracy.

Although most GP research for ST and MT for SE or ME has been applied to diploid species, a recent study by Ortiz *et al*. (2022) demonstrated the increase in prediction accuracy of ST-ME over the ST-SE genomic-estimated breeding values for several tetrasomic potato (*Solanum tuberosum* L.) breeding clones and released cultivars for various traits evaluated in several sites for 1 year. Ortiz *et al*. (2022) considered 4 dosages of marker alleles (A) pseudo-diploid; (B) additive tetrasomic polyploidy, and (C) additive-nonadditive tetrasomic polyploidy, and B + C dosages together in the genome-based prediction models using the conventional linear GBLUP (GB) and the nonlinear GK for ST-SE and ST-ME together. Results show that GK did not show any clear advantage over GB, and ST-ME had prediction accuracy estimates higher than those obtained from ST-SE. The model with GB was the best method in combination with the marker structures C or B + C for predicting most of the tuber traits. Most of the traits gave relatively high prediction accuracy under this combination of marker structure C or (B + C) and methods GB and GK combined with ST-ME including the GE model.

Based on the above considerations, and the need to extend research on GP to polysomic polyploid plant species, the main objectives of this research were to investigate ST vs MT for ME (GE) models using trial data from 3 locations [namely Helgegården (HEL), Mosslunda (MOS), and Umeå (UM)] over 2 years (2020, 2021) of 253 potato cultivars and breeding clones, which were also included by Ortiz *et al*. (2022). In this study, we will use only the genomic relationship matrix obtained from the additive-nonadditive tetrasomic polyploidy (C), because using this genomic relations matrix in terms of GP accuracy was found to be one with the best GP accuracy (Ortiz *et al*. 2022). This research investigated the GP of 4 genome-based prediction models including either Hadamard or Kronecker product matrices for assessing GE: (1) the conventional reaction norm model incorporating GE with Hadamard product (Jarquín *et al*. 2014) (*M1*); (2) GE model considering covariances between environments, similar to the model employed by Burgueño *et al*. (2012) or the GE with Kronecker product (*M2*); (3) GE model 2 including a random vector that attempts to more efficiently utilize the environmental covariances as in Cuevas *et al*. (2017) or a GE with Kronecker product (*M3*); and (4) an MT model with GE as in Montesinos-López *et al*. (2022), but including a GE model that joins Hadamard and Kronecker products (*M4*). Several prediction problems were analyzed for the GP accuracy of each of the 4 models. We investigated the prediction set of locations in the year 2021 from locations in the year 2020 using the 4 GP models combined with 2 of the prediction sets (100 and 70%) and predicting ST and MT.

## Materials and methods

### Phenotypic data

The MT experiments included 256 potato breeding clones and cultivars in trials at HEL, MOS, and UM. Their list is provided by Ortiz *et al*. (2022)Supplementary Table 1 (https://hdl.handle.net/11529/10548617). The breeding clones are in at least the fourth generation (T_4_) of selection by Svensk potatisförädling of the Swedish University of Agricultural Sciences (Ortiz *et al*., 2020), while the cultivars are a sample of those released and grown in Europe during the last 200 years. HEL and MOS are near Kristianstad (56°01′46″N 14°09′24″E, Skåne, southern Sweden), while UM (63°49′30″N 20°15′50″E) is in the north of Sweden.

An incomplete block design (simple lattice) with 2 replications of 10 plants each was the field layout for the field trials across testing sites. Fungicides were only used in HEL to avoid late blight caused by the oomycete *Phytophthora infestans* throughout the growing season, thus allowing tuber yield potential to be estimated at this site. Crop husbandry was used for potato farming at each site.

Total tuber yield per plot (kg), tuber weight by size (<40, 40–50, 50–60, >60 mm; kg), while tuber flesh starch was measured as a percentage based on specific gravity after harvest. Reducing sugars in the tuber flesh after harvest was determined using potato glucose strip tests (Mann *et al*. 1991). Host plant resistance to late blight was evaluated using the area under the disease progress curve in MOS.

### Genotypic data

After sampling using 4 leaf punches for each of the 256 breeding clones and cultivars included in the experiments, the materials were sent by AgriTech—Intertek ScanBi Diagnostics (Alnarp, Sweden) to Diversity Array Technology Pty Ltd (ACT, Australia) for targeted genotyping following a genotype-by-sequencing approach (https://www.diversityarrays.com/technology-and-resources/targeted-genotyping/). More than 2,000 single-nucleotide polymorphisms (SNP) were used for genotyping. They derived mostly from SolCAP SNPs based on chromosome positions and MAF > 0.05 in germplasm from the Centro Internacional de la Papa (CIP, Lima, Perú) and the United States of America. According to Selga *et al*. (2021), such a number of SNPs seems to be enough for researching GEBVs without losing information. Although there were very few missing genotyping data (0.1%), one breeding clone (97) and 2 cultivars (“Leyla” and “Red Lady”) were not included further in the analysis because they were lacking enough SNP data.

### Computing the genomic relationship matrix

We briefly described the method used for codifying the molecular ***X*** matrix proposed by Slater *et al*. (2016) and used one of the options used by Ortiz *et al*. (2022) in the genomic-enabled prediction models.

### Full tetrasomic including additive and nonadditive effects

For coding matrix ***X***, according to Slater *et al*. (2016), we considered additive and nonadditive effects in a full tetrasomic polyploid assuming each genotype, has its own effect. In this case, there were 5 possible (AAAA, AAAB, AABB, ABBB, BBBB) effects per SNP marker, coding 0, and 1, for the absence or presence of the genotype, respectively, in each of the 5 cases. For each SNP marker, exists 5 columns on ***X*** coding the presence or absence of the genotype. Then the genomic relationship between individuals *j*, *k* was computed as

$$K_{\;jk}=\frac{\frac{1}{M}\sum_{i=1}^{M}\;(x_{\;ji}-p_{i})(x_{ki}-p_{i})}{\;p_{i}(1-p_{i})}$$

where *M* was the number of markers × 5, *x*_*ji*_ represents the code of the absence or presence of the genotype from column *i*th of individual *j*th, and *p*_*i*_ is the frequency of each genotype, i.e. the frequency in each column. To compute the diagonal of this matrix, we used:

$$K_{\;jj}=1+\frac{1}{M}\frac{\sum_{i=1}^{M}\;(x_{\;ji}^{2}-2p_{i}x_{\;ji}+p_{i}^{2})\;}{\;p_{i}(1-p_{i})}$$

### Statistical models

#### ST conventional reaction norm model including GE (model 1, M1)

The standard reaction norm model incorporating GE (Jarquín *et al*. 2014), as shown below, explains the variation of the observations of a ST in each of the *m* environments (ME) represented by the vector $y=(y_{1}^{\prime },\ldots ,y_{i}^{\prime },\ldots y_{m}^{\prime }{)}^{\prime }$ by estimating each mean of the environment observations ***μ***_*E*_, plus the prediction of the main genetic effects ***g*** and the prediction of the interaction random effects G × E represented by vector ***ge***, the unexplained variation or random errors are represented by vector ***ɛ***.

$$y=Z_{E}\mu_{E}+g+ge+\varepsilon \;(1)$$

where ***y*** is a column vector of size *n*_*T*_ × 1, Considering *n*_*T*_ as the sum of the number of observations in each environment. The incidence matrix ***Z***_*E*_ relates the observations to the mean of the environments. The random genetic vector of main effects ***g*** follows a multivariate normal distribution $N(0,\;\sigma_{g}^{2}$$Z_{g}{KZ}_{g}^{\prime })$ where $\sigma_{g}^{2}$ is the variance component of ***g***, ***Z***_*g*_ is an incidence matrix that relates the observations with the ***K*** matrix of genomic relations between the clones. In our study, ***K*** was computed as previously indicated for the case of a full tetrasomic genomic relationship matrix. The random vector of interaction effects ***ge*** follows a multivariate normal distribution $N(0,\;\sigma_{ge}^{2}\;Z_{g}{KZ^{\prime }}_{g}\;\#\;Z_{E}{EZ}_{E}^{\prime }),$ where $\sigma_{ge}^{2}$ is the variance component, # denotes the Hadamard product, and ***E*** is a matrix of relationship between environments (in our case, an identity matrix is considered) such that $Z_{E}{EZ}_{E}^{\prime }$ is a block diagonal matrix with 1 s for all pairs of observations in the same environment and 0 s otherwise. This implies that the estimation of the effects ***ge*** is independent in each environment. Random errors ε are considered with homogeneous variance, that is, $\varepsilon ∼N(0,\;\sigma_{\varepsilon }^{2}I)$. This model is flexible because it allows predicting different numbers of clones in different environments or even predicting the entire environment. However, when the correlations between the environments are not positive, the GE model with the Hadamard product does not explain the phenotype variation well enough (Lopez-Cruz *et al*. 2015), because the model does not incorporate genomic covariances between environments.

#### ST GE (ST-ME) model considering covariances between environments (model 2, M2)

Based on Burgueño *et al*. (2012), the GP model including GE considered the genomic covariances between environments to attempt improving the GP accuracy of unobserved environments. In *M2*, we considered only one trait (ST) and MEs, but the main effect of genomic and the GE interaction effects are modeled jointly by using a single vector ***u*** assuming a multivariate normal distribution that considers the genomic covariances between environments. One form of this model is

$$y=Z_{E}\mu_{E}+u+\varepsilon \;\;(2)$$

where the genetic random effects can be modeled as a normal distribution $u∼N(0,\;U_{E}⊗K),$ where ***U***_*E*_ is a matrix of genomic covariances between the environments of size *m × m* to be estimated, and ⨂ indicates the Kronecker product. The random errors are modeled as ε∼N(0,Σ⊗I), where matrix Σ is a diagonal matrix of size *m × m*, that has on its diagonal the variances of the errors between environments to be estimated, and ***I*** is the identity matrix of order *n*_*L*_ × *n*_*L*_ (Cuevas *et al*. 2017), where *n*_*L*_ denotes the number of lines or clones in each environment (for balance data). Although model *M2* is powerful when considering the genetic covariances between environments, it cannot predict full environments because it does not have a way of estimating the corresponding genomic covariances of those environments in the training sites with those in the testing sites where no data have been collected.

#### ST GE model (ST-ME) with an extra random vector to better account for variance across environments (model 3, M3)

Cuevas *et al*. (2017) showed that adding a random vector to *M2* to account for the cultivar variation across environments that was accounted for by vector ***u***, could increase the prediction accuracy. Here, we considered a ST measured in different environments (ME) to construct and add a random vector ***f*** to *M2*, that is

$$y=Z_{E}\mu_{E}+u+f+\varepsilon$$

Then a random vector ***f*** is added that is independent from ***u***, and ε, and that has a normal distribution $f∼N(0,\;F_{E}⊗I)$, where ***F***_*E*_ is a matrix of environmental covariances of size *m × m* to be estimated, ⨂ indicates the Kronecker product, and matrix ***I*** represents the identity matrix. Note that the vector ***f*** allows predicting the nonadditive effects (or a proportion) for possible covariances that were not modeled in ***K***. Model *M3*, like *M2*, allows improving the prediction accuracy of model *M1*, when the covariances (or correlations) of the observations between environments are negative or close to zero. Like *M2*, *M3* could not be used to predict complete environments because, technically, it could not estimate covariances between related environments with the environments to be predicted because of the lack of data on the environments to be predicted.

#### MT model with GE (model 4, M4) of MT-ME type

Note that *M2* could be adopted to be a single environment MT (MT-SE) as

$$y=Z_{T}\mu_{T}+u+\varepsilon$$

where the vectors ***Z***_*T*_***μ***_*T*_ are similar to those of M2, that is, the ***μ***_*T*_ is a vector that represents the means of the *t* traits, and the incidence matrix ***Z***_*T*_ relates the observations with the mean of the traits, but now the number of cultivars is the same for each trait so that if we order the phenotypic observations of the first trait, then the second trait and so forth, $y=(y_{1}^{\prime },\;\ldots \;,y_{i}^{\prime },\;\ldots \;y_{t}^{\prime }{)}^{\prime }=\left[\begin{array}{c} y_{1}\;\vdots \;y_{t} \end{array}\right]$; then the genetic random effects can be modeled as a normal distribution $u∼N(0,\;U_{T}⊗K),$ where ***U***_*T*_ is a matrix of genomic covariances between the traits of size *t × t* to be estimated, and ⨂ indicates the Kronecker product. The matrix ***K*** represents the relationships between the genotypes built with molecular markers. The random errors are modeled as ε∼N(0,Σ⊗I), where the diagonal matrix Σ is a matrix of size *t × t*, expressing the covariances of the errors to be estimated; and ***I*** is the identity matrix of order *n*_*L*_ × *n*_*L*_.

This model MT-SE can also be represented as a multiresponse model, that is, instead of representing the observations as a vector, they can be arranged in a matrix so that *M2* can be re-written as

$$Y=1_{n}\mu^{\prime }+u+\varepsilon \;\;(2a)$$

where ***Y*** is a matrix of order *n*_*L*_*× t* that represents the phenotypic values ordered in such a way that the columns contain the data for each trait and the rows contain the data for each line or genotype. The intercepts or means of each trait are represented by a vector ***μ*** of size *t ×* 1. The matrix of genetic random effects assumes that they follow a multivariate multiresponse normal distribution $u∼MN_{n_{L}\times t}(0,\;K,\;U_{T})$. The random errors assume a multivariate multiresponse normal distribution $\varepsilon ∼MN_{n_{L}xt}(0,\;I,\;\Sigma )$.

As already mentioned, when MT data are available, the models to be used are those that account for correlations between phenotypic traits because when the degree of correlation is moderate or large, this could increase the GP accuracy. The model, based on the Bayesian MT kernel of Montesinos-López *et al*. (2022), can be seen as the combination of the MT model 2*a* and the reaction norm G × E *M1* for ME. Then *M4* is represented as

(4) $$Y=1_{n_{T}}\mu^{\prime }+Z_{E}\mu_{E}+g+ge+\varepsilon \;$$

where the matrix ***Y*** is of size *n*_*T*_ × *t* ordered in such a way that the columns represent the phenotypic values of each of the *t* traits and the rows are the lines or genotypes, ordered first by environments, and then by lines. The vector ***μ*** is of size *t* × 1 and it represents the intercept or mean of each trait. The matrix ***Z***_*E*_ is an incidence matrix of the environments of size *n*_*T*_ × *m*, and ***μ***_*E*_ is a matrix of order *m × t* with the means of each environment in each trait. The matrix ***g*** is of order *n*_*T*_ × *t* and follows a normal distribution $g∼MN_{n_{T}\times t}(0,\;Z_{g}{KZ}_{g}^{\prime },\;U_{g})$, where ***Z***_*g*_ is an incidence matrix of the genotypes of order *n*_*T*_ × *n*_*L*_, ***K*** is the relationship matrix of the genotypes of size *n*_*L*_ × *n*_*L*_ and *U*_*g*_ is a variance–covariance matrix of main effects between the traits. The matrix ***ge*** is of order *n*_*T*_ × *t* and follows a normal distribution $ge∼MN_{n_{T}xt}(0,\;Z_{g}{KZ}_{g}^{\prime }\#\;Z_{E}Z_{E}^{\prime },U_{ge})$, where # is the Hadamard product and ***U***_*ge*_ is a variance–covariance matrix of interaction effects between the traits. Random errors are represented by the matrix ***ɛ*** of order *n*_*T*_ × *t* that follows a normal distribution $\varepsilon ∼MN_{n_{T}xt}(0,\;I,\;\Sigma_{t})$, where the identity matrix ***I*** is of dimension *n*_*T*_ × *n*_*T*_.

### Studying different models and cross-validation schemes to assess the accuracy of the GP prediction models

The GP accuracy of the different models can be assessed by means of several different validation schemes. The first validation scheme (predicts 100% of the cultivars next year) uses the traits from each of the 3 locations in 2020 (HEL, MOS, and UM) to predict all the values of the traits in each of the 3 locations in 2021 (HEL, MOS, and UM). The second validation scheme (predicts 70% next year) uses all the data from 2020 plus 30% of the value of the traits in 3 locations in 2021 to predict 70% (prediction set) of the value of the traits at the 3 locations in 2022; this second case was established with 10 groups or random samples.

**Fig. 1.**
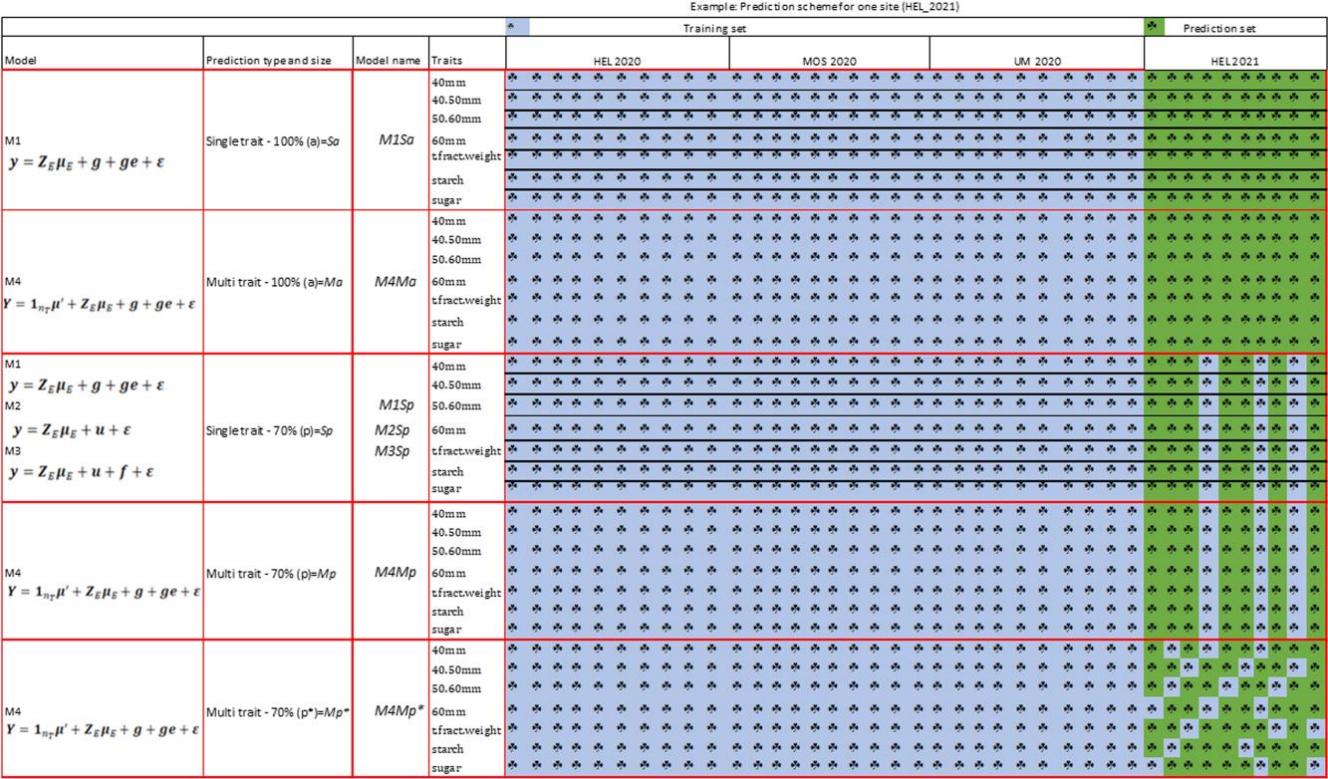
Hypothetical example with 10 cultivars for various models and sizes of prediction set (PS) for the GP of 7 potato traits at HEL in 2021 (PS) from training data observed at HEL, MOS, and Umeå (UM) in 2020. Models are *M1*–*M4* and PS are 100% or 70%. The 4 genome-based prediction models are *M1*: ST conventional reaction norm model incorporating GE; *M2*: ST GE model considering covariances between environments; *M3*: ST GE *M2* extended to include a random vector that more efficiently utilizes the environmental covariances; and *M4*: MT model with GE. The prediction sets contain all cultivars or a random cross-validation where 70% are predictive at HEL 2021. Red lines delineated the 5 random partition combinations and black lines identified ST GP and the absence of black lines identified MT GP.

The acronyms used for identifying models *M1*–*M4*, ST (*S*) or MT (*M*) and prediction set comprising the prediction of all cultivars in each location during 2021 (*a*), or the prediction of a percentage of cultivars in each location during 2021 (*p*) are given in Table 1. A graphical explanation of the different combinations of models (*M1*–*M*4), considering 2 prediction sets (100 and 70%), and ST or MT cross-validation schemes for assessing the GP prediction accuracy of the models is shown in Fig. 1 for 10 hypothetical cultivars evaluated in HEL, MOS, and UM in 2020 to predict HEL in 2021. The only MT model is *M4*, whereas ST models are *M1*, *M2*, and *M3*.

**Table 1.** Acronyms for models, traits, and scenarios for predicting locations in 2021.

Acronyms	Model	Single-trait or multitrait	Prediction set for 2021
*M1Sa*	*M1*	Single trait (*S*)	All cultivars (*a*)
*M4Ma*	*M4*	Multitrait (*M*)	All cultivars (*a*)
*M1Sp*	*M1*	Single trait (*S*)	Cultivar sample (70%) (*p*)
*M2Sp*	*M2*	Single trait (*S*)	Cultivar sample (70%) (*p*)
*M3Sp*	*M3*	Single trait (*S*)	Cultivar sample (70%) (*p*)
*M4Mp*	*M4*	Multitrait (*M*)	Sample (70%) of some cultivars that were not observed in any of the traits (*p*)
M4Mp*	*M4*	Multitrait (*M*)	Sample (70%) of some cultivars that were observed in some traits but not in other traits (p*)

As shown in Fig. 1, the first cross-validations refer to 2 cases including models *M1* and *M4* for predicting all the values (100%) for each trait in location HEL 2021 using as a training set all the values for each trait in each location from 2020. Model *M1* is an ST (traits are separated by black lines), whereas *M4* is an MT model (traits are not separated). For these 2 cases, the given acronyms join (1) the model (*M1–M4*), (2) the ST or MT (*S* or *M*) prediction, and (3) include the prediction of all (100%) the lines in HEL 2021 and denoted by “*a*,” that is, *M1Sa* and *M4Ma*. The third and fourth cross-validation schemes delineated by red lines included models *M1*, *M2*, and *M3* for ST and model *M4* for MT, and they predict 70% of the values of each trait in HEL 2021, using as training set values of the trait in each location from 2020, but also adding 30% of the values from HEL 2021 to the prediction set in the training set. As already mentioned, this prediction of 70% is performed 10 times using the 10 random samples for extracting 30% of the values of the prediction set (2021) and adding them to the training set (2020). The same 10 random samples were used for comparing the GP accuracy of the 4 models.

The names of each of these model-prediction types and sizes are *M1Sp*, *M2Sp*, *M3Sp*, and *M4Mp* where the letter “*p*” refers to the percentage of the prediction set (70%). Note that for these 4 cases, 3 cultivars (out of 10) are missing in all the traits (Fig. 1). The fifth cross-validation scheme had MT *M4* that predicts 70% of the cultivars in HEL in 2021 for all traits, but now, the cross-validations between the traits and locations for HEL 2021 are different from those in the previous case (*M4Mp*) where some cultivars are observed in some traits and locations but not observed in other traits and locations. This cross-validation scheme is refereed to M4Mp*. Note that in this case, some cultivars are missing in some traits but not in other traits; for example, cultivars 1, 2, and 3 are not observed for the weight of tubers below 40 mm, but are observed for the weight of 40 − 50 mm tubers (Fig. 1).

### Measures of prediction accuracy

We used 2 metrics for comparing the genomic-enabled prediction accuracy of the different models (*M1*, *M2*, *M3*, and *M4*). One metric is the Pearson correlation coefficient (COR) between the observed and predicted values, whereas the second metric is the prediction mean squared error (PMSE) of the different prediction models.

## Results

In this study, we used 3 genomic models (*M1*, *M2*, and *M3*) that predict one ST and various environments (ME). The first model *M1* is the ST conventional reaction norm model that considers the genomic main effect and interaction effects with homogeneous variance for the environmental random errors. Model *M2* considers together the genomic effects and heterogenous environmental variance error. Note that model *M3* adds a random vector to *M2* with the aim of capturing some nonadditive genetic effects that were not previously explained. Finally, model *M4* includes MTs as multiresponse and MEs.

Two main prediction scenarios were analyzed: (1) use models *M1* and *M2* to predict all potato cultivars for each of the 3 locations in 2021 where the training were the locations in 2020, and (2) using all 4 models to predict 70% of the potato cultivars of each location in 2021, and incorporating 30% of the prediction set in the training set. Note that we used acronyms to identify the model (*M1–M4*), the ST (*S*) or MT (*M*), and the size of the prediction set, all cultivar (*a*) or a percentage (*p*) (Table 1, Fig. 1).

**Fig. 2.**
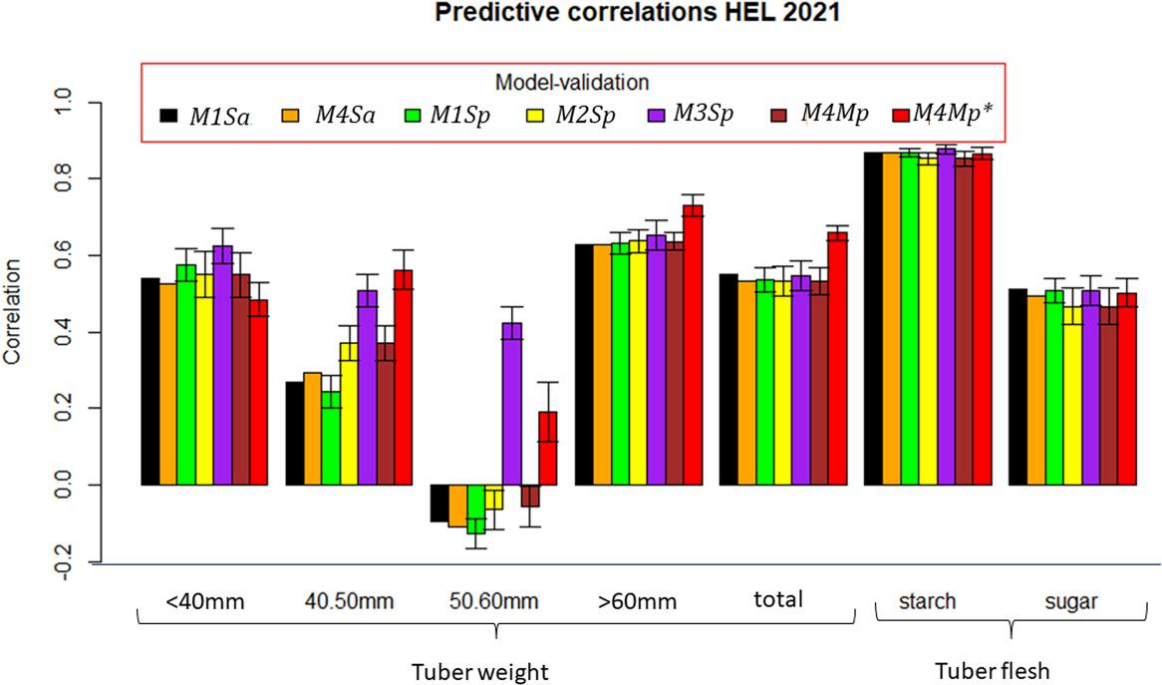
Correlation between observed and predicted values for 7 traits at HEL (2021) using 7 methods. One standard deviation with respect to the mean of the correlation is denoted by the bar. Model/methods *M1Sa* and *M4Sa* do not have standard deviations. *M1Sa* is the prediction accuracy from model *M1* (ST conventional reaction norm model incorporating GE) when predicting 100% of each trait in 2021). *M4Ma* is the prediction accuracy from model *M4* (MT model with GE) when predicting 100% of each trait in 2021. *M1Sp* is the prediction accuracy from model *M1* when predicting 70% of each trait in 2021. *M2Sp* is the prediction accuracy from model *M2* when predicting 70% of each trait in 2021. *M3Sp* is the prediction accuracy from model *M3* when predicting 70% of each trait in 2021. *M4Mp* is the prediction accuracy from model *M4* when predicting 70% of each trait in 2021. M4Mp* is the prediction accuracy from model *M4* when predicting 70% of each trait in 2021, in which, some percent of cultivars are observed in some traits.

**Fig. 3.**
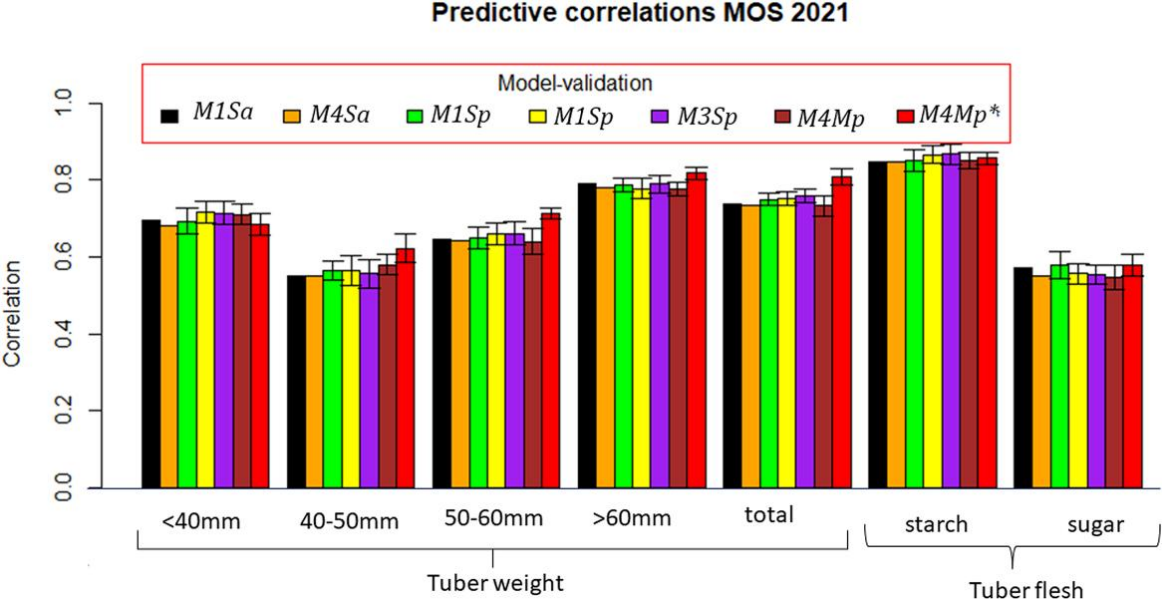
Correlation between observed and predicted values for 7 traits at MOS (2021) using 7 methods. One standard deviation with respect to the mean of the correlation is denoted by the bar. Methods *M1Sa* and *M4Sa* do not have standard deviations. *M1Sa* is the prediction accuracy from model *M1* (ST conventional reaction norm model incorporating GE) when predicting 100% of each trait in 2021. *M4Ma* is the prediction accuracy from model *M4* (MT model with GE) when predicting 100% of each trait in 2021. *M1Sp* is the prediction accuracy from model *M1* when predicting 70% of each trait in 2021. *M2Sp* is the prediction accuracy from model *M2* when predicting 70% of each trait in 2021. *M3Sp* is the prediction accuracy from model *M3* when predicting 70% of each trait in 2021. *M4Mp* is the prediction accuracy from model *M4* when predicting 70% of each trait in 2021. M4Mp* is the prediction accuracy from model *M4* when predicting 70% of each trait in 2021, in which, some cultivars are observed in some traits.

**Fig. 4.**
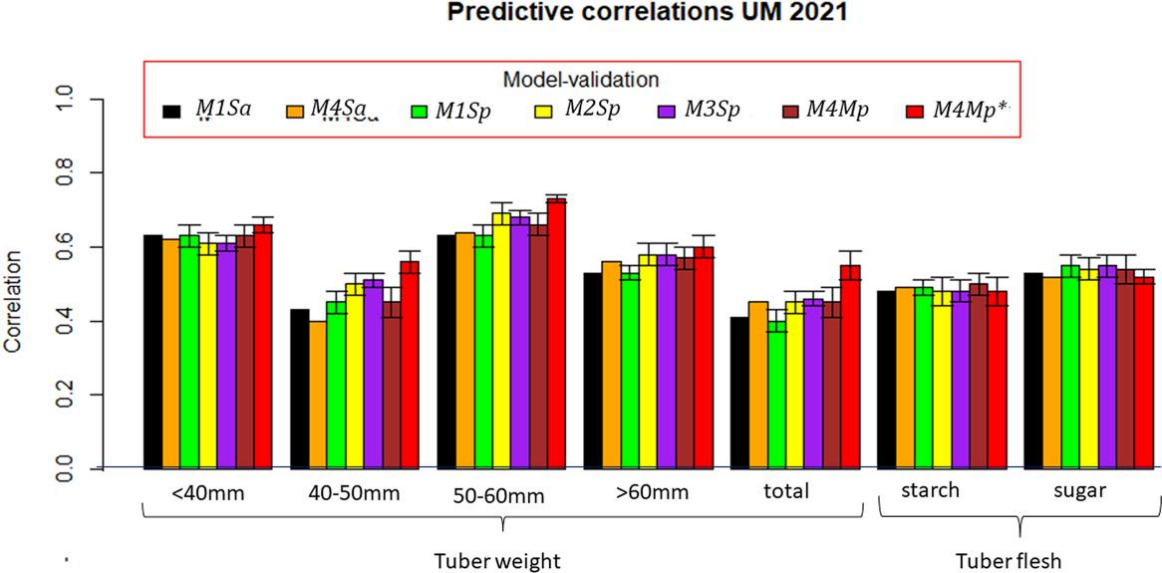
Correlation between observed and predicted values for 7 traits at Umeå (2021) using 7 methods. One standard deviation with respect to the mean of the correlation is denoted by the bar. Methods *M1Sa* and *M4Sa* do not have standard deviations). *M1Sa* is the prediction accuracy from model *M1* (ST conventional reaction norm model incorporating GE) when predicting 100% of each trait in 2021. *M4Ma* is the prediction accuracy from model *M4* (MT model with GE) when predicting 100% of each trait in 2021. *M1Sp* is the prediction accuracy from model *M1* when predicting 70% of each trait in 2021. *M2Sp* is the prediction accuracy from model *M2* when predicting 70% of each trait in 2021. *M3Sp* is the prediction accuracy from model *M3* when predicting 70% of each trait in 2021. *M4Mp* is the prediction accuracy from model *M4* when predicting 70% of each trait in 2021. M4Mp* is the prediction accuracy from model *M4* when predicting 70% of each trait in 2021, in which, some cultivars are observed in some traits.

Phenotypic correlations were computed for traits in each location (HEL, MOS, and UM) in 2021 (prediction set, PS) with those traits observed in the locations of the previous year (HEL, MOS, and UM in 2020; Table 2). The PS contains 7 traits (5 tuber weight traits and 2 tuber flesh quality characteristics) in each of the 3 locations of 2021 using the locations and traits of the previous year, 2020. The *M1–M4* ST or MT prediction models for predicting all cultivars or a proportion of cultivars (all cultivars or 70%) are combined in the following acronyms: *M1Sa*, *M4Ma*, *M1Sp*, *M2Sp*, *M3Sp*, *M4Mp*, and M4Mp* (Tables 3–5 and Figs. 2–4).

**Table 2.** Phenotypic correlations of each trait at HEL in 2021 with each trait at HEL 2020, MOS 2020, and Umeå (UM) 2020.

Site-year	Traits						
	Weight of tubers					Tuber flesh	
	<40 mm	40–50 mm	50–60 mm	> 60mm	Total	Starch	Sugar
HEL 2021							
HEL 2020	0.62	0.60	0.24	0.68	0.64	0.89	0.36
MOS 2020	0.36	0.20	−0.16	0.49	0.48	0.80	0.30
UM 2020	0.43	−0.05	−0.25	0.51	0.39	0.78	0.43
MOS 2021							
HEL 2020	0.65	0.49	0.56	0.73	0.64	0.83	0.39
MOS 2020	0.64	0.50	0.61	0.74	0.74	0.89	0.36
UM 2020	0.55	0.28	0.45	0.62	0.52	0.72	0.41
UM 2021							
HEL 2020	0.49	0.04	0.42	0.53	0.38	0.48	0.31
MOS 2020	0.49	0.30	0.47	0.40	0.29	0.40	0.33
UM 2020	0.57	0.51	0.67	0.57	0.46	0.46	0.46

Phenotypic correlations of each trait oat MOS 2021 with each trait at HEL 2020, MOS 2020, and UM2020. Phenotypic correlations of each trait at UM 2021 with each trait at HEL 2020, MOS 2020, and UM 2020.

**Table 3.** Predictive correlations (CORs) and PMSE for predicting 7 traits at HEL in 2021 for 4 models (*M1*, *M2*, *M3*, *M4*) combined with 100% or 70% cross-validation.

Model name	Prediction accuracy measures	Traits 2021						
		Tuber weight					Tuber flesh	
		<40 mm	40–50 mm	50–60 mm	>60 mm	Total	Starch	Sugar
*M1Sa*	COR	0.539	0.269	−0.097	0.627	0.551	0.868	0.511
	PMSE	0.552	1.630	6.271	17.040	12.020	1.601	0.730
*M4Ma*	COR	0.525	0.292	−0.111	0.628	0.533	0.867	0.493
	PMSE	0.388	1.702	5.049	16.940	12.600	1.640	0.804
*M1Sp*	COR (mean)	0.576	0.244	−0.127	0.632	0.537	0.868	0.508
	COR (SD)	0.043	0.043	0.038	0.028	0.031	0.010	0.031
	PMSE (mean)	0.143	0.975	4.785	16.300	11.878	1.582	0.799
	PMSE (SD)	0.022	0.058	0.570	0.708	0.677	0.088	0.035
*M2Sp*	COR (mean)	0.549	0.370	−0.065	0.637	0.533	0.852	0.466
	COR (SD)	0.060	0.045	0.051	0.029	0.039	0.016	0.048
	PMSE (mean)	0.226	1.433	5.615	16.732	11.948	1.620	0.799
	PMSE (SD)	0.028	0.086	0.676	0.730	0.677	0.185	0.038
*M3Sp*	COR (mean)	0.623	0.508	0.424	0.651	0.548	0.877	0.508
	COR (SD)	0.046	0.041	0.042	0.039	0.039	0.012	0.040
	PMSE (mean)	0.076	0.716	3.112	13.460	11.812	1.459	0.746
	PMSE (SD)	0.012	0.061	0.345	1.346	1.146	0.106	0.054
*M4Mp*	COR (mean)	0.549	0.370	−0.057	0.636	0.533	0.852	0.467
	COR (SD)	0.058	0.047	0.052	0.023	0.034	0.018	0.047
	PMSE (mean)	0.142	0.863	4.317	16.833	12.242	1.650	0.810
	PMSE (SD)	0.020	0.060	0.614	0.701	0.644	0.103	0.036
M4Mp*	COR (mean)	0.484	0.562	0.191	0.730	0.658	0.866	0.502
	COR (SD)	0.044	0.050	0.077	0.029	0.021	0.016	0.037
	PMSE (mean)	0.154	0.684	3.858	12.279	10.000	1.607	0.796
	PMSE (SD)	0.014	0.125	0.411	1.084	0.760	0.125	0.048

*M1Sa* is the prediction accuracy from model *M1* [ST conventional reaction norm model incorporating GE] when predicting 100% of each trait in 2021; *M4Ma* is the prediction accuracy from model *M4* (MT model with GE) when predicting 100% of each trait in 2021; *M1Sp* is the prediction accuracy from model *M1* when predicting 70% of each trait in 2021; *M2Sp* is the prediction accuracy from model *M2* (ST GE model considering covariances between environments) when predicting 70% of each trait in 2021; *M3Sp* is the prediction accuracy from model *M3* (ST GE *M2* extended to include a random vector that more efficiently utilizes the environmental covariances) when predicting 70% of each trait in 2021; *M4Mp* is the prediction accuracy from model *M4* when predicting 70% of each trait in 2021, M4Mp* is the prediction accuracy from model *M4* when predicting 70% of each trait in 2021, in which, some cultivars are observed in some traits. When predicting 70%, the mean and the standard deviations (SDs) from the 10-fold cross-validation are given in parentheses.

**Table 4.** Predictive correlations (CORs) and PMSE for predicting 7 traits at MOS in 2021 for 4 models (*M1*, *M2*, *M3*, *M4*) combined with 100% or 70% cross-validation.

Model name	Prediction accuracy measures	Traits 2021						
		Tuber weight					Tuber flesh	
		<40 mm	40–50 mm	50–60 mm	>60 mm	Total	Starch	Sugar
*M1Sa*	COR	0.694	0.550	0.647	0.791	0.739	0.847	0.572
	PMSE	0.112	0.587	0.949	1.600	3.700	2.050	0.890
*M4Ma*	COR	0.680	0.551	0.641	0.779	0.734	0.848	0.550
	PMSE	0.256	0.595	1.420	0.940	3.756	2.100	0.840
*M1Sp*	COR (mean)	0.693	0.564	0.648	0.786	0.749	0.851	0.578
	COR (SD)	0.032	0.025	0.028	0.018	0.016	0.028	0.034
	PMSE (mean)	0.113	0.583	0.949	1.599	3.400	1.991	0.877
	PMSE (SD)	0.010	0.034	0.100	0.123	0.165	0.251	0.040
*M2Sp*	COR (mean)	0.717	0.564	0.660	0.777	0.750	0.866	0.556
	COR (SD)	0.029	0.038	0.029	0.026	0.018	0.024	0.026
	PMSE (mean)	0.075	0.591	0.919	1.701	3.459	1.777	0.807
	PMSE (SD)	0.006	0.044	0.097	0.164	0.264	0.222	0.063
*M3Sp*	COR (mean)	0.714	0.557	0.660	0.790	0.758	0.867	0.553
	COR (SD)	0.029	0.037	0.030	0.023	0.018	0.026	0.025
	PMSE (mean)	0.075	0.595	0.920	1.605	3.335	1.757	0.817
	PMSE (SD)	0.005	0.042	0.095	0.145	0.221	0.234	0.058
*M4Mp*	COR (mean)	0.710	0.580	0.640	0.776	0.732	0.851	0.546
	COR (SD)	0.026	0.027	0.033	0.019	0.027	0.021	0.032
	PMSE (mean)	0.077	0.578	1.023	1.804	3.405	2.345	0.904
	PMSE (SD)	0.004	0.066	0.048	0.131	0.232	0.166	0.037
M4Mp*	COR (mean)	0.684	0.622	0.711	0.817	0.808	0.856	0.579
	COR (SD)	0.029	0.036	0.014	0.016	0.020	0.017	0.028
	PMSE (mean)	0.105	0.546	0.804	1.410	2.782	1.890	0.881
	PMSE (SD)	0.004	0.066	0.048	0.131	0.232	0.166	0.037

*M1Sa* is the prediction accuracy from model *M1* [ST conventional reaction norm model incorporating GE] when predicting 100% of each trait in 2021. *M4Ma* is the prediction accuracy from model *M4* (MT model with GE-like) when predicting 100% of each trait in 2021. *M1Sp* is the prediction accuracy from model *M1* when predicting 70% of each trait in 2021. *M2Sp* is the prediction accuracy from model *M2* (ST GE model considering covariances between environments) when predicting 70% of each trait in 2021. *M3Sp* is the prediction accuracy from model *M3* (ST GE *M2* extended to include a random vector that more efficiently utilizes the environmental covariances) when predicting 70% of each trait in 2021; *M4Mp* is the prediction accuracy from model *M4* when predicting 70% of each trait in 2021, M4Mp* is the prediction accuracy from model *M4* when predicting 70% of each trait in 2021, in which, some cultivars are observed in some traits. When predicting 70%, the mean and the standard deviations (SDs) are given from the 10-fold cross-validation in parentheses.

**Table 5.** Predictive correlations (COR) and PMSE for predicting 7 traits at UM in 2021 for 4 models (*M1*, *M2*, *M3*, *M4*) combined with 100% or 70% cross-validation.

Model name	Prediction accuracy measures	Traits 2021						
		Tuber weight					Tuber flesh	
		<40 mm	40–50 mm	50–60 mm	>60 mm	Total	Starch	Sugar
*M1Sa*	COR	0.626	0.425	0.625	0.527	0.411	0.479	0.529
	PMSE	0.540	1.127	0.925	0.715	6.680	6.220	0.817
*M4Ma*	COR	0.617	0.400	0.641	0.563	0.446	0.488	0.515
	PMSE	0.544	1.220	0.885	0.703	5.742	5.860	0.824
*M1Sp*	COR(mean)	0.633	0.445	0.629	0.534	0.404	0.487	0.545
	COR (SD)	0.034	0.034	0.031	0.024	0.033	0.021	0.027
	PMSE (mean)	0.537	1.125	0.922	0.674	6.909	6.128	0.802
	PMSE (SD)	0.052	0.071	0.088	0.106	0.600	0.554	0.049
*M2Sp*	COR (mean)	0.605	0.502	0.688	0.578	0.450	0.481	0.544
	COR (SD)	0.025	0.025	0.025	0.032	0.031	0.036	0.032
	PMSE (mean)	0.556	1.054	0.796	0.674	5.705	5.896	0.744
	PMSE (SD)	0.080	0.078	0.067	0.054	0.352	0.459	0.054
*M3Sp*	COR (mean)	0.605	0.512	0.682	0.581	0.463	0.483	0.550
	COR (SD)	0.024	0.019	0.024	0.031	0.022	0.029	0.034
	PMSE (mean)	0.557	1.042	0.809	0.671	5.581	5.879	0.741
	PMSE (SD)	0.082	0.064	0.070	0.056	0.398	0.457	0.053
*M4Mp*	COR (mean)	0.627	0.451	0.663	0.573	0.449	0.496	0.537
	COR (SD)	0.035	0.043	0.028	0.029	0.035	0.025	0.020
	PMSE (mean)	0.535	1.137	0.875	1.257	6.375	5.982	0.792
	PMSE (SD)	0.056	0.098	0.088	0.119	0.646	0.555	0.063
M4Mp*	COR (mean)	0.662	0.558	0.732	0.603	0.551	0.482	0.519
	COR (SD)	0.020	0.033	0.012	0.030	0.036	0.044	0.019
	PMSE (mean)	0.428	0.949	0.710	1.064	5.324	5.854	0.851
	PMSE (SD)	0.027	0.065	0.035	0.084	0.533	0.371	0.077

*M1Sa* is the prediction accuracy from model *M1* [ST conventional reaction norm model incorporating GE] when predicting 100% of each trait in 2021. *M4Ma* is the prediction accuracy from model *M4* (MT model with GE) when predicting 100% of each trait in 2021. *M1Sp* is the prediction accuracy from model *M1* when predicting 70% of each trait in 2021. *M2Sp* is the prediction accuracy from model *M2* (ST GE model considering covariances between environments) when predicting 70% of each trait in 2021. *M3Sp* is the prediction accuracy from model *M3* (ST GE *M2* extended to include a random vector that more efficiently utilizes the environmental covariances) when predicting 70% of each trait in 2021. *M4Mp* is the prediction accuracy from model *M4* when predicting 70% of each trait in 2021, M4Mp* is the prediction accuracy from model *M4* when predicting 70% of each when some cultivars are observed in some traits. When predicting 70% the mean and the standard deviations (SDs) from the 10-fold cross-validation are given in parentheses.

### GP of traits in HEL 2021, MOS 2021, UM 2021

Table 2 lists the phenotypic correlations for each trait measured at HEL, MOS, and UM in 2021 with those of 2020. These correlations are certainly related to the prediction accuracy estimates shown in Tables 3–5 and displayed in Figs. 2–4; i.e. the mean prediction accuracy estimates are higher for the cases when the phenotypic correlations between years were higher as was the case for starch. In these cases, where the phenotypic correlations between traits for the 2 years were high, the mean prediction accuracy between the models did not show significant differences. Furthermore, when the phenotypic correlations between locations are moderate, as for example for reducing sugars, the accuracy of the model's predictions did not show significant differences (Figs. 2–4). On the contrary, when the phenotypic correlations were negative or near zero, as for example, for the weight of 50–60 mm tubers for HEL 2021 (Fig. 2), the prediction accuracy estimates were low in the models except for *M3Sp* and M4Mp*. Similar results were observed for the weight of 40–50 mm tubers at HEL 2021 (Fig. 2) and UM 2021 (Fig. 4), where models *M3Sp* and M4Mp* had better predictions than models *M1Sa* and *M1Sp*, which showed lower predictions when the phenotypic correlations were close to zero or negatives.

Overall, the model showing the best prediction accuracy was M4Sp* closely followed by model *M3Sp*. However, the differences were higher when the phenotypic correlations between the locations were near zero or negative.

### GP of traits in HEL 2021

Genomic predictions including all cultivars in HEL 2021 were the best for tuber flesh starch in all the models, whose GP accuracy estimates were above 0.85 (Table 3 and Fig. 2). Most of the 4 models had a very similar GP accuracy for starch; i.e. ranging from 0.852 (*M2Sp* and *M4Mp*) to 0.877 (*M3Sp*) (Table 3, Fig. 2).

The second trait with an important GP accuracy shown by most of the models was the weight of 60 mm tubers. The MT model predicting a proportion of cultivars (M4Mp*) had the highest prediction accuracy (0.730, Table 3) and a ST conventional reaction norm model for predicting that all cultivars (*M1Sa*) had the lowest GP accuracy (0.627). The weight of tubers below 40 mm and the total tuber weight had a very similar GP accuracy except for the MT model M4Mp*, which was the worst model for the weight of tubers below 40 mm but the best model for total tuber weight. Excluding M4Mp*, the predictions ranged from 0.525 (<40 mm, *M4Ma*) to 0.623 (<40 mm *M3Sp*) for both traits. The best predictive model was *M3Sp* for the weight of tubers below 40 mm and *M1Sa* for total tuber weight (Fig. 2). Weight with 40–50 mm tubers and 50–60 mm tubers had the lowest prediction accuracy for most models except *M3Sp* (Fig. 2). Comparing the models with ST and MT, *M3Sp* was the best ST model for tuber weight below 40 mm and between 50 and 60 mm, and tuber flesh starch, whereas M4Mp* was best for weights of 40–50 mm and above 60 mm tubers, as well as for the total tuber weight.

In summary, prediction of the 7 traits at HEL in 2021 shows that traits with a higher phenotypic correlation between location HEL 2021 and those at HEL, MOS, and UM in 2020 are tuber flesh starch and most of the tuber weights (except the weight of 50–60 mm tubers). In terms of GP accuracy, the MT model predicting 70% of the cultivars for some traits while observing others (M4Mp*) was the best for weight 40–50 mm tubers or above 60 mm tubers, and total tuber weight, and very similar to those for tuber flesh starch. Model *M3Sp* was the best GP for the weight of tubers below 40 mm and 50–60 mm, as well as for tuber flesh starch.

### GP of traits in MOS 2021

The phenotypic correlation of traits measured in location MOS in 2020–2021 is given in Table 2. For all the traits, phenotypic correlations between traits in MOS for 2021 and 2020 were higher than those between MOS 2021 and the 2 other locations (HEL and UM) in 2020. Tuber flesh starch had the highest phenotypic correlation between MOS 2021and HEL, MOS, and UM 2020 (0.83, 0.89, and 0.72, respectively) followed by the weight of tubers above 60 mm (0.73, 0.74, and 0.62, respectively), total tuber weight (0.64, 0.74, and 0.52, respectively), and the weight of tubers below 40 mm (0.65, 0.64, and 0.55, respectively).

Overall GP accuracy in MOS 2021 was higher than in HEL 2021. Tuber flesh starch was the best-predicted trait for all the models with GP accuracy below 0.85 (Table 4 and Fig. 3). Most of the 4 models showed a very similar GP accuracy for tuber flesh starch but ST *M2* and *M3* predicting 70% of the cultivars (*M2Sp* and *M3Sp*) were the best genomic predictors, with 0.866 and 0.867, respectively. Models *M1* and *M4* predicting all potato cultivars (*M1Sa* and *M4Ma*) were slightly below in terms of prediction accuracy (0.847 and 0.848, respectively).

The second trait with important GP accuracy shown by most of the models was the weight of tubers above 60 mm with M4Mp* with an accuracy of 0.817, followed by*M1Sa* having an accuracy of 0.791, followed by *M3Sp* with 0.790 (Table 4). Overall, the total tuber weight irrespective of size, ranked third based on GP accuracy, with model M4Mp* having a prediction accuracy of 0.808, followed by *M3Sp* with 0.758 prediction accuracy, followed by *M2Sp* (0.750). The weight of tubers below 40 mm had relatively high GP accuracy, with models *M2Sp* and *M3Sp* being the best with 0.717 and 0.714 of GP accuracy, respectively. Finally, the weight of 50–60 mm tubers had lower prediction accuracy than the previously mentioned traits, with the best predictor models being M4Mp*—whose GP accuracy was 0.711—followed by *M2Sp* and *M3Sp* (GP accuracy = 0.660).

The GP accuracy estimates for the 7 traits in MOS during 2021 were slightly higher than those at HEL 2021. The traits with higher phenotypic correlations between MOS 2021 and those at HEL, MOS, and UM in 2020 were tuber flesh starch, the weight of tubers above 60 mm and below 40 mm, total tuber weight, and the weight of 50–60 mm tuber. Overall, the best models for predicting most of the 7 traits were the ST models *M2* and *M3* predicting 70% of the potato cultivars in each location (*M3Sp* and *M2Sp*), except for traits such as the weight of 50–60 mm and above 60 mm tubers, and the total tuber weight in which, the MT model M4Mp* was the best GP model.

### GP of traits in location UM 2021

Table 2 lists the phenotypic correlation of traits measured at UM in 2020–2021. For all the traits, the phenotypic correlations between traits in UM for 2021 and 2020 are higher than those between UM 2021 and other locations (HEL and MOS) in 2020. The traits with the highest phenotypic correlation between UM 2021 and HEL, MOS, and UM 2020 were the weight of 50–60 mm, below 40 mm, and above 60 mm tubers, followed by tuber flesh starch.

Overall, the GP accuracy in UM 2021 was lower than those of HEL and MOS in 2021. The weight of 50–60 mm and below 40 mm tubers were the best-predicted traits for all the models in UM 2021 (Table 5 and Fig. 4). The best GP model for all the traits, except reducing sugars and starch in the tuber flesh, was M4Mp*. ST and MT models predicting 70% of the cultivars (*M3Sp* and *M4Mp*) had the best GP accuracy for predicting traits of tuber flesh sugar and starch, respectively.

Most of the 4 models showed similar GP accuracy for these 2 traits, but *M2Sp* had a GP accuracy of 0.688 for the weight of 50–60 mm tubers, and model *M4Mp* had an accuracy of 0.633 for the weight of tubers below 40 mm. Models *M2Sp* and *M3Sp* had a GP accuracy of around 0.578 for the weight of tubers above 60 mm that ranked third on overall GP accuracy (Table 5) followed by tuber flesh starch, with model *M3Sp* being the best with (prediction accuracy = 0.483), followed by *M2Sp* (0.481).

The GP accuracy of the 7 traits at UM in 2021 showed lower accuracy in 2021 than at HEL and MOS in 2021. Traits with higher phenotypic correlations between UM 2021 and those at HEL, MOS, and UM in 2020 are the weight of tubers with 50–60 mm, below 40 mm, and above 60 mm. However, the best model for predicting the majority of the 7 traits was the MT predicting 70% with 30% of the testing added to the training (M4Mp*) followed by models *M4Mp* for tuber flesh starch and *M3Sp* for tuber flesh sugar.

## Discussion

The integration of GS and GP to develop modern cultivars faster than the conventional breeding method is necessary for increasing genetic gains and facing the changes in climate that are currently affecting agriculture. Thus, a better and an efficient integration of new methods including GS with increased GP accuracy, rapid cycle GS, high throughput phenotyping, and the use of appropriate environmental covariables is an urgent area of research (Crossa *et al*. 2021). The integration and exploitation of several big data sets are necessary, and the use of appropriate statistical machine-learning models has become important for modern breeding.

### Prediction accuracy of models for ST and MT, cross-validation method, and proportion of the prediction set

When performing research on GS and GP accuracy, several problems become important; one is the inclusion of statistical machine-learning methods and models that include GE interaction. Another problem to be assessed is the addition of several traits for prediction rather than only one trait, and another issue is the methods used for comparing the GP accuracy of several traits using several models and various possible cross-validation schemes to develop a GP accuracy metric. Several options exist for investigating the GS accuracy for predicting the breeding value of cultivars that have been genotyped with genome-wide molecular markers. One scenario is predicting the performance of a proportion of cultivars (e.g. 70%) that have not yet been observed in any of the testing environments (usually location-year combinations); another option is to predict all cultivars (i.e. 100%) observed in all the environments except one (leave one environment out). Another scenario is predicting cultivars that were observed in some environments but not in others.

In this study, predictions for these scenarios have been done using ST (*M1*, *M2*, and *M3*) and MT (*M4*) models. These ST and MT models combined with different prediction testing scenarios are described in Table 1 and graphically displayed in a small example in Fig. 1, where several proportions of the PS have been combined with the 4 different models. We included the predictions of all cultivars in 1 entire site-year combination or the prediction of a proportion of cultivars (70%) using the other 30% as TS together with the previous year. We found that for the majority of the traits in each location-year combination to be predicted (HEL, MOS, UM in 2021) *M4* (MT), with a proportion of potato cultivars evaluated (30%) in some location-year combinations M4Mp* (Fig. 1) but not observed in other location-year combinations, was found to be the best predictive model, usually followed by ST models *M3Sp* and *M2Sp*.

Results of this study demonstrate that for predicting traits in HEL 2021 using all environments in 2020, the superiority of the MT prediction method M4Mp* over the mean GP accuracy of the other 6 prediction methods including ST and MT for predicting the entire PS (100%) or 70% for traits tuber weights 40–50 mm, above 60 mm and total in this location were 65, 14, and 24%, respectively. However, this superiority of the MT over ST methods was not so when comparing *M4Ma* or *M4Mp* with other ST methods, especially for *M3Sp* for traits tuber weight <40 mm, 50–60 mm, and tuber flesh starch. Results for predicting traits at MOS in 2021 using all environments in 2020 show the superiority of the MT prediction method M4Mp* for 4 tuber weight traits and 1 tuber flesh quality characteristic over all the other 6 methods. The GP accuracy of method M4Mp* overcame the mean GP accuracy of all the other 6 methods by 10, 9, 4, 8, and 4% for the weight of 40–50 mm, 50–60 mm, above 60 mm tubers, total tuber weight and tuber flesh sugar, respectively. Similar results were obtained for the prediction of location UM in 2021 using the TS comprising HEL, MOS, and UM from 2020; the best GP accuracy method for all 5 tuber weight traits was method M4Mp* over the mean GP accuracy of all the other 6 methods by 7, 24, 12, 8, and 26% for tuber weights below 40 mm, 40–50 mm, 50–60 mm, above 60 mm and total tuber weight, respectively.

Previous research noticed variable prediction accuracy that depends on factors such as heritability of the trait, size of TP, relatedness of PS and TS, statistical machine-learning models, marker density, linkage disequilibrium, and the incorporation of GE interactions in the prediction models. In a recent article, Semagn *et al*. (2022) compared the predictive abilities of wheat cultivars that have not been evaluated for an ST, not evaluated for MTs (MT1), and evaluated for some traits but not others (MT2) using agronomy and disease traits. Note that the partition of Semagn's MT1 is similar to the partitions of *Sp* (*M1*, *M2*, and *M3*) and *Mp* (*M4*) in this study, whereas the partitions of Semagn's MT2 are similar to that of M4Mp*. Semagn *et al*. (2022) found that the GP accuracy of MT2 (method M4Mp* in this study) increased over ST and other model-partitions in all traits from 9 to 82%. This occurred because, under the prediction scheme MT2 of Semagn *et al*. (2022), it is possible to exchange information between traits like method M4Mp* that allows borrowing of information between traits and also between environments, and thus, to efficiently use the available information in one single model combined with an appropriate prediction scheme.

This demonstrated the high potential for improving prediction accuracies and the high potential of the MT models for improving prediction accuracy, thus offering researchers the opportunity to predict traits that were not observed, due to possible difficulties or because they are expensive to measure under certain environmental constraints (Semagn *et al*. 2022).

### Prediction accuracy of potato traits

Genomic prediction in potatoes is still in the early research stages before using it for routine breeding of this highly heterozygous tetrasomic polyploid tuberous crop with vegetative propagation (Ortiz *et al*. 2022, and references therein). The use of MT and ME models for GP in this research led to the highest accuracy for tuber yield and tuber flesh starch as per available literature. Tuber flesh starch, which is often estimated from specific gravity measurements, is a very highly heritable trait (Bradshaw 2021; Ortiz *et al*. 2021) that is affected very little by the GEs (Killick and Simmonds 1974), thus explaining the high prediction accuracy noted in this and research elsewhere. The high prediction accuracy noted in this, and previous research suggests that developing GEBV modeling in potatoes for tuber flesh starch does not require a very large training population, but it seems that just a few hundred (including both breeding clones and released cultivars that are relevant to the breeding program and covering a broad range of trait variation) may suffice.

Genotype × environment interactions may significantly affect tuber yield, but the use of ME GP allows identifying promising germplasm in both crossing blocks (Ortiz *et al*. 2022) in potato breeding. The significantly high correlations noted when using MT, ME modeling suggest that GP may also be useful for the potato cultivar development pipeline even when using small breeding populations (Selga *et al*. 2022). Every year, F_1_ seeds (resulting from crossing heterozygous parents) are planted in individual pots in a greenhouse, and one tuber (the best in size) for each plant is taken at harvest. Thus, thousands of tubers derived from these F_1_ hybrid seeds are produced for further field testing in single plant plots during the first year. At harvest, all plants are dug up to assess their tuber number, size, shape, color, appearance, and health, which are used as the selection criteria for obtaining the next breeding generation for further testing the next year. After selection in early clonal generations [first (T_1_), second (T_2_), and often third (T_3_)], the aim is to have about a few dozens for field testing from the fourth generation onward and ending with a few promising breeding clones after the seventh year of field testing and selection to include them in MT trials in the target population of environments. The GP accuracy over the 2 years within each site suggests that it will be possible to select (based on GEBV models) in early generation trials for each target population of environments. Furthermore, as per previous GP accuracy estimates (Ortiz *et al*. 2022; Selga *et al*. 2022) and these results, it seems that GEBV for selection will be useful from T_3_ onward, rather than in T_1_ or even in T_2_. Hence, as shown herein, genomic selection appears to be feasible in potato breeding when using elite-bred germplasm.

### Conclusion

The ST model *M3Sp* was the best genomic predicted, followed by *M1Sp* and *M1Sa* at HEL in 2021. In terms of MT GP accuracy, M4Mp* was the best for the weight of 40–50 mm and above 60 mm tubers, and total tuber weight irrespective of size, and very similar to tuber flesh starch. The GP accuracy of the 7 traits at MOS in 2021 indicated that the best models for predicting the majority of the 7 traits were ST *M3Sp* and *M2Sp*, except for the weight of 50–60 mm tubers, above 60 mm tubers, and total tuber weight, where the MT model M4Mp* was the best GP model. The traits with higher phenotypic correlations between location UM 2021 and those at HEL, MOS, and UM in 2020 are the weight of tubers with the following sizes: 50–60 mm, below 40 mm, and above 60 mm. The best model method for predicting the majority of the 7 traits was MT M4Mp* because it allows the exchange of information between traits and environments followed by *M3Sp* and *M2Sp,* which efficiently used information between environments. According to Cuevas *et al.* (2017), *M3Sp* producing better or similar GP accuracy than *M2Sp* was expected.

## Supplementary Material

jkac322_Supplementary_Data

## Data Availability

DNA marker and phenotypic data for each year within sites are stored at https://hdl.handle.net/11529/10548784. Supplemental material is available at *G3* online.

## Notes

*Communicating editor: J.L. Jannink*
