# Supplementary material for: Modeling genotype × environment interaction for single and multitrait genomic prediction in potato (Solanum tuberosum L.)
Source: G3 (Bethesda). 2022 Dec 8;13(2):jkac322. doi: 10.1093/g3journal/jkac322 (PMC9911059; doi:10.1093/g3journal/jkac322)
Supplement: jkac322_Supplementary_Data [file jkac322_supplementary_data.docx]

Supplementary Table S1. SLU’s Svensk potatisförädling breeding clones and cultivars available for growing in Sweden used for field trials in Helgegården, Mosslunda and Umeå

| Cultivar or Breeding Clone | Country | Released or Crossing Year |
| --- | --- | --- |
| Belinda | AUT | 1979 |
| Ditta | AUT | 1989 |
| Kerkauer Kipfler | AUT | N/A |
| Linzer Delikatess | AUT | 1975 |
| Afra | BRD | 1990 |
| Agila | BRD | 2006 |
| Agria | BRD | 1985 |
| Alexandra | BRD | 2008 |
| Allians | BRD | 2003 |
| Almonda | BRD | 2011 |
| Annalena | BRD | N/A |
| Antonia | BRD | 2008 |
| Baby Lou | BRD | N/A |
| Bellarosa | BRD | 2004 |
| Belmonda | BRD | 2010 |
| Birgit | BRD | 2009 |
| Blaue Annelise | BRD | N/A |
| Centifolia | BRD | 1919 |
| Cilena | BRD | 1981 |
| Concordia | BRD | 2008 |
| Corinna | BRD | N/A |
| Edison | BRD | N/A |
| Finka | BRD | 2001 |
| Gala | BRD | 2002 |
| Goldmarie | BRD | N/A |
| Granola | BRD | 1975 |
| Gunda | BRD | 1999 |
| Hansa | BRD | 1957 |
| Heiderot | BRD | 1977 |
| Jelly | BRD | 2002 |
| Jule | BRD | N/A |
| Julinka | BRD | N/A |
| Krone | BRD | 2002 |
| Laura | BRD | 1998 |
| Lea | BRD | N(A |
| Leyla | BRD | 1988 |
| Lilly | BRD | N/A |
| Linda | BRD | 1974 |
| Ludmilla | BRD | 2008 |
| Marena | BRD | 1995 |
| Merle | BRD | N/A |
| Nandina | BRD | 2010 |
| Natalia | BRD | N/A |
| Natascha | BRD | 2006 |
| Nicola | BRD | 1973 |
| Odenwälder Blaue | BRD | 1908 |
| Orla | BRD | 2001 |
| Otolia | BRD | N/A |
| Papageno | BRD | 2019 |
| Pocahontas | BRD | N/A |
| Princess | BRD | 1998 |
| Quarta | BRD | 1979 |
| Queen Anne | BRD | 2012 |
| Ragna | BRD | 1982 |
| Ramona | BRD | 1988 |
| Red Emmalie | BRD | N/A |
| Red Fantasy | BRD | 2006 |
| Red Lady | BRD | 2004 |
| Regina | BRD | 2009 |
| Rodriga | BRD | 2005 |
| Rosara | BRD | 1990 |
| Rossini | BRD | N/A |
| Salad Blue | BRD | N/A |
| Selma | BRD | 1972 |
| Sieglinde | BRD | 1935 |
| Solara | BRD | 1989 |
| Solist | BRD | 1999 |
| Sunshine | BRD | N/A |
| Talent | BRD | 2006 |
| Torenia | BRD | N/A |
| Venezia | BRD | 2009 |
| Verdi | BRD | 2003 |
| Anuschka | BRD | 2004 |
| Belana | BRD | 2000 |
| Quadriga | BRD | 2005 |
| Blaue St. Galler | CH | N/A |
| Madeira | CH | N/A |
| Adretta | DDR | 1975 |
| Karlena | DDR | 1988 |
| Aeggeblomme | DK | N/A |
| Folva | DK | 1989 |
| Tammiston | FIN | 1930 |
| Timo | FIN | 1975 |
| Altesse | FRA | 2000 |
| Amandine | FRA | 1993 |
| Anais | FRA | 1997 |
| Aura | FRA | 1951 |
| Belle de Fontenay | FRA | 1885 |
| Blue Belle | FRA | 2008 |
| Charlotte | FRA | 1981 |
| Cherie | FRA | 1997 |
| Cheyenne | FRA | N/A |
| Dalida | FRA | N/A |
| Gaiane | FRA | 2018 |
| Glorietta | FRA | 2020 |
| Gwenne | FRA | 2011 |
| Juliette | FRA | 1997 |
| La Ratte | FRA | 1965 |
| Maestro | FRA | 2001 |
| Marine | FRA | 1994 |
| Monique | FRA | 2020 |
| Vitelotte | FRA | 1815 |
| Yona | FRA | 2008 |
| Zoe | FRA | 2018 |
| Anya | GB | 1996 |
| Arran Pilot | GB | 1930 |
| Arran Victory | GB | 1918 |
| British Queen | GB | 1984 |
| Casa Blanca | GB | 2010 |
| Emily | GB | N/A |
| Golden Wonder | GB | 1906 |
| Highland Burgundy Red | GB | 1930 |
| Inca Bella | GB | 2010 |
| International Kidney | GB | 1879 |
| King Edward | GB | 1902 |
| Kingsman | GB | N/A |
| Maris Bard | GB | 1972 |
| Maris Peer | GB | 1962 |
| Marys Rose | GB | 2001 |
| Mayan Gold | GB | 2001 |
| Mayan Rose | GB | N/A |
| Pink Fir Apple | GB | 1850 |
| Red Duke of York | GB | N/A |
| Rocket | GB | 1987 |
| Sarpo Una | GB | 2010 |
| Shetland Black | GB | N/A |
| Swift | GB | 1994 |
| Valor | GB | 1993 |
| Foxton | GB | 1981 |
| Sarpo Mira | HUN | 2003 |
| Cara | IRL | 1973 |
| Colleen | IRL | 1991 |
| Galactica | IRL | 2003 |
| Rooster | IRL | 1990 |
| Setanta | IRL | 2004 |
| Raudar | IS | 1800 |
| 7 FOUR 7 | NDL | 2014 |
| A | NDL | 2018 |
| Allstar | NDL | 2015 |
| Alouette | NDL | 2014 |
| Annabelle | NDL | 2001 |
| Anouk | NDL | 2014 |
| Apache | NDL | 1986 |
| Arielle | NDL | 1999 |
| Arrow | NDL | 2004 |
| Arsenal | NDL | 2009 |
| Asperges | NDL | N/A |
| Asterix | NDL | 1991 |
| Avenue | NDL | 2010 |
| Bintje | NDL | 1910 |
| Bionica | NDL | 2008 |
| Carolus | NDL | 2012 |
| Challenger | NDL | 2008 |
| Colomba | NDL | 2011 |
| Connect | NDL | 2012 |
| Dartiest | NDL | 2012 |
| Desiree | NDL | 1962 |
| Eigenheimer | NDL | 1885 |
| Fontane | NDL | 1999 |
| Frieslander | NDL | 1990 |
| Hind | NDL | 2018 |
| Inova | NDL | 1999 |
| Jazzy | NDL | 2010 |
| Kuras | NDL | 1996 |
| Labella | NDL | 2008 |
| B | NDL | 2019 |
| C | NDL | 2007 |
| D | NDL | 2010 |
| E | NDL | 2008 |
| Lady Christl | NDL | 1996 |
| F | NDL | 1996 |
| G | NDL | 1988 |
| Laperla | NDL | 2010 |
| Marabel | NDL | 1993 |
| Marilyn | NDL | 2006 |
| Masai | NDL | 2014 |
| H | NDL | 2001 |
| Minerva | NDL | 1988 |
| Montana | NDL | 1998 |
| Monte Carlo | NDL | 2009 |
| I | NDL | 2007 |
| Nofy | NDL | 2017 |
| J | NDL | 2007 |
| Purple Rain | NDL | 2019 |
| Record | NDL | 1932 |
| Saprodi | NDL | 2014 |
| Serum Star | NDL | N/A |
| Sevilla | NDL | 2018 |
| K | NDL | 2005 |
| Sunita | NDL | 2014 |
| Taisiya | NDL | 2011 |
| Tarzan | NDL | 1989 |
| Theresa | NDL | 1979 |
| Triplo | NDL | 2000 |
| Twister | NDL | 2017 |
| Ultra | NDL | 1999 |
| Marius | NOR/POL | 1893 |
| 0003022 | SLU | 2000 |
| 0101011 | SLU | 2001 |
| 0502047 | SLU | 2005 |
| 1201001 | SLU | 2012 |
| 1209001 | SLU | 2012 |
| 1211009 | SLU | 2012 |
| 1212001 | SLU | 2012 |
| 1213008 | SLU | 2012 |
| 1312011 | SLU | 2013 |
| 1314013 | SLU | 2013 |
| 1314015 | SLU | 2013 |
| 1326009 | SLU | 2013 |
| 1337013 | SLU | 2013 |
| 1337015 | SLU | 2013 |
| 1337017 | SLU | 2013 |
| 1342004 | SLU | 2013 |
| 1402001 | SLU | 2014 |
| 1402003 | SLU | 2014 |
| 1402009 | SLU | 2014 |
| 1410004 | SLU | 2014 |
| 1410005 | SLU | 2014 |
| 1414011 | SLU | 2014 |
| 1415001 | SLU | 2014 |
| 1415003 | SLU | 2014 |
| 1419002 | SLU | 2014 |
| 1419004 | SLU | 2014 |
| 1419006 | SLU | 2014 |
| 1419009 | SLU | 2014 |
| 1419010 | SLU | 2014 |
| 1429006 | SLU | 2014 |
| 1433004 | SLU | 2014 |
| 1433005 | SLU | 2014 |
| 1433006 | SLU | 2014 |
| 1438004 | SLU | 2014 |
| 1442007 | SLU | 2014 |
| 1452001 | SLU | 2014 |
| 2-IV-4 | SLU CWR | N/A |
| 2-IV-6 | SLU CWR | N/A |
| N5-18 | SLU CWR | N/A |
| 16 | SLU* |  |
| 96 | SLU* |  |
| 97 | SLU* |  |
| 107 | SLU* |  |
| 121 | SLU* |  |
| 127 | SLU* |  |
| 131 | SLU* |  |
| 188 | SLU* |  |
| Blå Mandel | SWE | N/A |
| Blaue Schweden | SWE | 1800 |
| Mandel | SWE | N/A |
| Maria | SWE | 1972 |
| Perlo | SWE | 2007 |
| Röda krokar | SWE | 1800 |
| Sparris | SWE | N/A |
| Tärendö | SWE | N/A |
| Magda | TCH | 2000 |
| Early Puritan | USA | 1988 |
| Early Rose | USA | 1897 |
| Purple Majesty | USA | N/A |

AUT = Austria, BRD = Germany, CH = Switzerland, DDR = former East Germany, DK = Denmark, FIN = Finland, FRA = France, GB = Great Britain, IRL = Ireland, IS = Iceland, NDL = The Netherlands, NOR/POL = Norway/Poland, SWE = Sweden, TCH = Czechia, USA = United States of America, N/A = unavailable, SLU CWR = SLU’s crop wild relative-derived breeding clone, SLU* = SLU’s chip potato breeding clone
